# Elevated l-threonine is a biomarker for Lassa fever and Ebola

**DOI:** 10.1186/s12985-020-01459-y

**Published:** 2020-11-26

**Authors:** Trevor V. Gale, John S. Schieffelin, Luis M. Branco, Robert F. Garry, Donald S. Grant

**Affiliations:** 1 Department of Microbiology and Immunology, Tulane University, 1430 Tulane Avenue, JBJ568, New Orleans, LA 70112 USA; 2 Sections of Infectious Disease, Departments of Pediatrics and Internal Medicine, School of Medicine, Tulane University, New Orleans, LA USA; 3 Zalgen Labs, LLC, Germantown, MD USA; 4 Viral Hemorrhagic Fever Program, Kenema Government Hospital, Kenema, Sierra Leone; 5 Ministry of Health and Sanitation, Freetown, Sierra Leone; 6 Present Address: Ansun Biopharma, San Diego, CA 92121 USA

**Keywords:** Lassa fever, Ebola, Viral hemorrhagic fevers, Liquid Chromatography Mass Spectrometry, l-Threonine, Metabolomics

## Abstract

**Background:**

Lassa fever and Ebola are characterized by non-specific initial presentations that can progress to severe multisystem illnesses with high fatality rates. Samples from additional subjects are examined to extend and corroborate biomarkers with prognostic value for these diseases.

**Methods:**

Liquid Chromatography Mass Spectrometry metabolomics was used to identify and confirm metabolites disrupted in the blood of Lassa fever and Ebola patients. Authenticated standards are used to confirm the identify of key metabolites.

**Results:**

We confirm prior results by other investigators that the amino acid l-threonine is elevated during Ebola virus infection. l-Threonine is also elevated during Lassa virus infection. We also confirmed that platelet-activating factor (PAF) and molecules with PAF moiety are reduced in the blood of patients with fatal Lassa fever. Similar changes in PAF and PAF-like molecules were not observed in the blood of Ebola patients.

**Conclusions:**

Metabolomics may provide tools to identify pathways that are differentially affected during viral hemorrhagic fevers and guide development of diagnostics to monitor and predict outcome.

## Background

Select features within the metabolome may serve as biomarkers for disease severity/progression/outcome and lend themselves to the design of prognostic methods for viral diseases, such as Lassa fever and Ebola. We have previously characterized endogenous small molecules with prognostic value originating from the blood of febrile patients triaged to the Lassa fever ward in Kenema, Sierra Leone [1]. In an extraordinary multi-omics study Eisfeld and coworkers [2] demonstrated that levels of serum l-threonine were elevated in the blood of patients with Ebola, another severe viral hemorrhagic fever (VHF). Prior research has shown that l-threonine has significance as a biomarker in infectious disease and inflammation. Previously, l-threonine has been reported as a biomarker for both tuberculosis [3] and malaria [4]. Our prior study identified multiple unknown metabolites as potential biomarkers of acute Lassa fever [1]. Inspired by the results of Eisfeld and coworkers [2] we corroborate l-threonine as a biomarker of acute Lassa fever. We also contrast levels of other select metabolites, including plate-activating factor (PAF) and PAF-like molecules, between Lassa fever and Ebola patients. Authenicate standards were used to confirm the identity of l-threonine and selected PAF-like molecules.

## Methods

### Human subjects

The Tulane University Institutional Review Board and the Sierra Leone Ethics and Scientific Research Committee approved this project. Patients were referred to the Kenema Government Hospital (KGH) Lassa Ward from the hospital’s general ward or from regional health centers on the basis of suspicion of Lassa fever. Patients who met the case definition of Lassa fever as defined by Khan et al. [5] were admitted and cared for by the ward’s trained staff. After the initial cases of Ebola were detected, patients were referred if they presented with an illness that met the World Health Organization case definition for Ebola. We obtained samples using the collection and processing protocols at Kenema Government Hospital under the emergency-response guidelines established by the Sierra Leone Ministry of Health and Sanitation. Diagnostic tests for the presence of Ebola virus (EBOV) were performed on site by means of quantitative reverse-transcriptase–polymerase chain reaction assays with the use of the SuperScript III One-Step RT-PCR System with Platinum Taq DNA Polymerase (Life Technologies).

### Serum processing for metabolomics analyses

Small blood volumes (approximately 5 mL) for serum separation were collected from patients presenting to KGH with febrile illnesses that met preclinical criteria of suspected Lassa fever or Ebola. Patient samples received a coded designation and were collected in serum vacutainer tubes. Blood samples were allowed to coagulate for 20 min at room temperature. Serum was separated from coagulated blood by centrifugation (200×*g*, 20 min at room temperature). For subjects for which there was excess serum not needed for clinical evaluations, aliquots of the serum fraction were stored in cryovials at − 20 °C prior to processing for metabolite analysis.

Serum metabolite analysis was performed as previously described [1]. Briefly, serum samples were depleted of protein by addition to one part sera (100 μL) of 4 parts ice-cold methanol (400 μL), the mixture was vortexed vigorously for 10 s, and incubated 1 h at − 20 °C followed by centrifugation at 14,000×*g*, 15 min, 4 °C. The supernatant was collected and transferred to a new, sterile vial and dried under vacuum. The resultant smal l-molecule containing pellets were stored in desiccated, sealed containers and shipped to Tulane University where they were gamma-irradiated. Small molecule containing pellets were dissolved in a solution of 95:5 water:acetonitrile transferred to autosampler vials, and held at − 20 °C or 4 °C immediately prior to analysis. All reagents utilized were high-pressure liquid chromatography (HPLC) grade.

### Liquid Chromatography Mass Spectrometry

The Liquid Chromatography Mass Spectrometry (LCMS) method was performed as previously described with minor changes [1]. Briefly, detection of metabolites was performed via HPLC separation with ESI–MS (electrospray mass spectrometry) detection. HLPC was performed with an aqueous norma l-phase, hydrophilic interaction chromatography HPLC column: a Cogent Diamond Hydride Type-C column with 4 μm particles and dimensions of 150 mm length and 2.1 mm diameter or an Agilent Zorbax 300-SB-C18 column with 3.0 μm particles and dimensions of 150 mm length and 0.3 mm diameter was used with an Agilent 1290 HPLC system (Agilent Technologies, Santa Clara, CA). The column were maintained at 60 °C with a flow rate of 900 μL/min. Chromatography was as follows: solvent consisted of H_2_O with 0.1% (v/v) formic acid for channel “A” and acetonitrile with 0.1% formic acid for channel “B”. Following column equilibration at 98% B, the sample was injected via autosampler, and the column was flushed for 2.0 min to waste. From 2.0 min to 14.5 min, the gradient was linearly ramped from 98 to 65% B. From 14.5 min to 16.0 min, the gradient was ramped from 65 to 25% B. From 14.5 to 18.0 min the column was held at 25% B, and from 18.0 to 18.2 min the gradient was ramped from 25 to 98% B. From 18.2 to 20.0 min the column was re-equilibrated with 98% B. An Agilent 6538 Quad-Time of Flight with dua l-electron spray ion source mass spectrometer was used for all analyses. Resolution was approximately 20,000 and accuracy was 1 ppm. Source parameters: drying gas 12 L/min, nebulizer 60 psi, capillary voltage 3500 V, capillary exit 100 V. Spectra were collected in positive mode from 50 to 1700 m/z at a rate of 1 Hz.

### Molecular standards

Authenticated standards of synthetic platelet-activating factor (PAF) C-16 (#878110) and lysoPAF C-16 (#878119) at a concentration of 5 mg/mL in chloroform were purchased from Avanti Polar Lipids (Birmingham, AL). l-threonine (T8625) was purchased from Sigma-Aldrich. The molecules were diluted in 95:5 water:acetonitrile solution and analyzed with the identical method for metabolite detection.

### Data analysis and visualization

Raw spectral data in.d format where uploaded to XCMS Online (Versions 2.3.0 or 2.2.3) and processed as pairwise comparisons using parameters optimized for data acquired with UPLC on an Agilent 6538 MS.

### Statistics and machine learning

Statistical analyses were carried out using the R statistical software package or Graphpad Prism. Multiple comparisons were performed by Analysis of Variance (ANOVA); *p* < 0.5 was considered significant. Raw mass spectral intensity values and a unique identifier for specific spectral features were extrapolated from XCMS output and compiled into.csv file types.

## Results

### Characteristics of a cohort of subjects presenting to Kenema Government Hospital with Lassa fever or Ebola

A panel of 50 serum samples from febrile patients triaged to a ward for the care of suspected Lassa fever patients was assembled to corroborate earlier observations of biomarkers of poor outcome in Lassa fever. Serum samples were drawn upon admittance and in all but two instances diagnostic tests were performed within 24 h. Twenty-two subjects tested negative for Lassa virus (LASV) by all diagnostic tests and were classified as febrile non-Lassa. Subjects with a positive diagnosis for Lassa fever were subdivided into patients that were discharged (n = 12) and those that succumbed to infection (n = 16). Gender and age data were available for 48 of the 50 samples. Subjects were 54% female (26/48) with a mean age of 25.0 (years, range < 1–60). There were 17 and 10 female and male LASV positive samples, respectively. Nine females and 6 males succumbed to Lassa fever with an average age of 21.5 (years, range < 1–38) and an average time from symptom onset to death of 7.75 (days, 5–16). There were no significant differences between the distribution of LASV positive subjects or mortality between female and male subjects. A panel of serum samples derived from KGH collected during the 2015 EBOV outbreak in West Africa were also analyzed. Twelve febrile subjects tested negative for Lassa virus (LASV) and EBOV by all diagnostic tests and were classified as febrile non-Ebola. Twenty-nine subjects tested positive for EBOV at the time of hospitalization and were classified as Ebola positive.

### l-Threonine is elevated in VHF patients

**Fig. 1:**
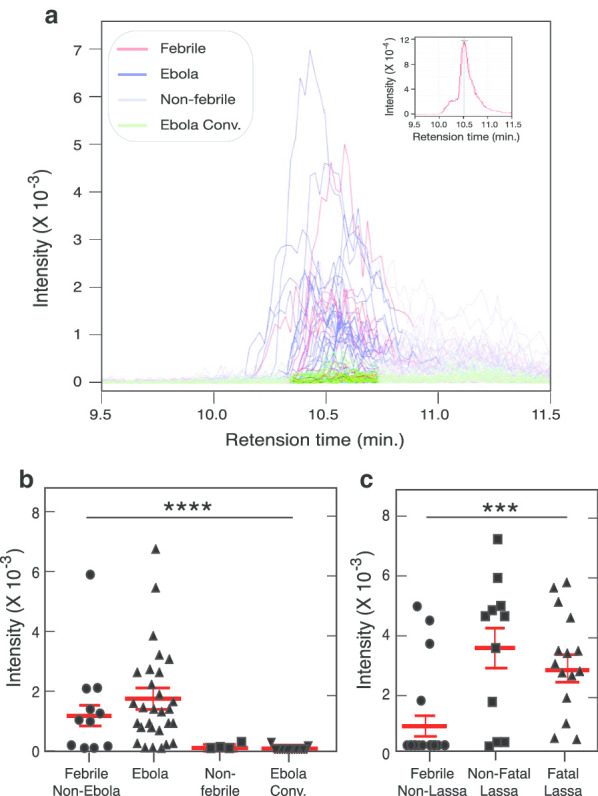
Levels of l-threonine in blood of viral hemorrhagic fever patients. **a** Intensity versus retention time plots for the H^+^H_2_O^−^ adduct of l-threonine in the blood of febrile patients without Ebola, Ebola patients, non-febrile patients and Ebola convalescent patients. Inset shows the Intensity versus retention time plot of an authentic l-threonine standard. **b** Levels of l-threonine in samples from febrile patients that tested negative for Ebola (n = 12) were compared to samples from patients with Ebola (n = 29), non-febrile controls (n = 4) or Ebola convalescent patients (n = 27). **c** Levels of l-threonine in samples from febrile patients that tested negative for Lassa fever (n = 22) were compared to samples from patients with non-fatal (n = 12) or fatal Lassa fever (n = 16). Signal intensity of serum threonine values are presented as mean and SEM. Significance levels (*p* values from one way ANOVA) are ***p < 0.0005 and ****p < 0.0001

Previously several unknown molecular features were identified in the blood of acutely ill Lassa fever patients [1]. Based on results from Eisfeld et al. [2] authentic standards were used to identify certain of the unknown molecules as adducts of l-threonine (Fig. 1). Two features that were significantly elevated and detected as mass-to-charge ratio (*m/z*) 102.0537 and 119.0800 at identical retention times [rt = 15.95]. These features have now been identified as the H^+^H_2_O^−^ and NH_4_^+^H_2_O^−^ adducts of l-threonine. l-threonine was significantly elevated in specimens from Ebola patients (Fig. 1a,b). In contrast, l-threonine was not elevated in the blood of convalescent Ebola patients or blood samples from non-febrile controls. Likewise, l-threonine was significantly elevated in specimens from Lassa fever patients compared to febrile subjects without Lassa fever (Fig. 1c). No significant different was observed between Lassa fever subjects that succumbed to Lassa fever and Lassa fever survivors. Most febrile patients that presented to the VHF Ward at KGH and tested negative for Ebola or Lassa fever had low blood levels of l-threonine. However, a subset of febrile patients in both the Ebola (Fig. 1b) and Lassa cohorts (Fig. 1c) had elevated blood l-threonine. These results show that elevated l-threonine may be a common marker of acute VHFs as patients positive for both EBOV and LASV infection have significant elevation of this compound in the blood across spatially and temporally distinct disease outbreaks.

### Platelet-activating factor and platelet-activating factor like-lipids are decreased in subjects with fatal Lassa fever

**Fig. 2:**
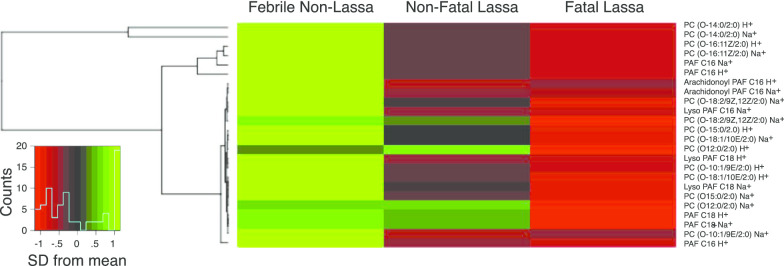
Cluster analysis of selected serum lipids in subjects with different Lassa virus serostatus and outcomes. The heat map represents levels of 24 putatively identified platelet activating factor (PAF) or PAF-like molecules. The inset scale represents the number of standard deviations (SD) from the mean value of the row

Platelet activity is depressed during Lassa fever, particularly in terminal patients [6, 7]. Twenty-four PAF or PAF-like molecules were putatively identified and expressed at variable levels in the serum of febrile patients presenting to KGH (Fig. 2). The cluster analysis indicated that nearly all PAFs or PAF-like molecules were present in lower amounts in the serum of patients with fatal Lassa fever than in patients that survived the acute infection (nonfatal Lassa fever). Non-Lassa febrile illness patients had the highest overall levels of PAF or PAF-like molecules. We also positively confirmed the identity of PAF and lysoPAF with LCMS versus authenticated standards in the serum of patients with Lassa fever. PAF and PAF-like molecules are reduced Lassa fever patients that have a fatal outcome. These lipids were not observed to be similarly dysregulated in a limited cohort of serum samples of Ebola positive patients compared to convalescent Ebola and febrile non-Ebola patients (Additional file 1: Fig. S1).

## Discussion

A previously unknown molecule elevated in fatal, acute, and post-Lassa acute febrile patients has been identified as l-threonine. The serum of Ebola patients also has elevated levels of l-threonine. Results showing elevation of l-threonine in the blood of Lassa fever and Ebola patients confirms and extend results previously published by Eisfeld and coworkers [2]. These investigators demonstrated that levels of serum l-threonine, as well as a vitamin D binding protein, perfectly stratified Ebola patients by outcome, providing better predictive ability than viral load. Their results prompted us to use authentic standards to identity l-threonine as a Lassa fever biomarker. The use of authenic standards for selected PAF-like molecules also corroborates and improves the results of our prior metabolomic analyses [1].

Prior metabolomic studies have identified l-threonine as a marker of infection by several viral and nonviral pathogens [2–4]. The amniotic fluid of human cytomegalovirus infected (HCMV) women shows elevated levels of l-threonine compared to non-HCMV infected women [8]. No significance difference in vertical transmission of HCMV based on elevated l-threonine was observed. Glycine, serine, and threonine metabolic pathways were found to be altered in chikungunya or dengue patients [9]. In contrast to prior results in Ebola patients [2], l-threonine was elevated in both Lassa fever patients that succumbed to their illness as well as subjects that survived. Further studies are required to determine whether elevated serum l-threonine represents a general marker of VHF or other severe viral infections. We confirmed that PAF and molecules with PAF moiety are reduced in the blood of patients with fatal Lassa fever. However, similar changes were not observed in the blood of Ebola patients. Additional studies on the different roles for PAF and PAF-like molecules in severe viral diseases should be conducted.

## Conclusions

Metabolomics may provide tools to identify pathways that are differentially affected during VHFs. For patients suffering from VHF it would also be advantageous to have a measure of disease progression/severity, such as l-threonine levels, to predict outcomes several days prior to death. Coupling the detection and changes of these and other analytes with appropriate rapid diagnostics through disease progression may serve as a mechanism to monitor and predict outcome, ensuring scarce resources are allocated where needed most.

## Supplementary information

**Additional file 1: Figure S1**. Levels of selected platelet-activating factor and platelet-activating factor-like molecules in the blood of Lassa fever and Ebola patients.

## Data Availability

Data has been deposited in the XCMS Public archive (https://xcmsonline.scripps.edu/landing_page.php?pgcontent=mainPage) under the identifier: 1181466.

## Abbreviations

VHF: Viral hemorrhagic fever
KGH: Kenema Government Hospital
EBOV: Ebola virus
LASV: Lassa virus
*m/z*: Mass-to-charge ratio
HPLC: High-pressure liquid chromatography
LCMS: Liquid Chromatography Mass Spectrometry
HCMV: Human cytomegalovirus infected
PAF: Platelet-activating factor
ANOVA: Analysis of variance
VHFC: Viral Hemorrhagic Fever Consortium
