# Supplementary material for: Elevated l-threonine is a biomarker for Lassa fever and Ebola
Source: Virol J. 2020 Nov 26;17:188. doi: 10.1186/s12985-020-01459-y (PMC7690152; doi:10.1186/s12985-020-01459-y)
Supplement: Supplementary file 1 — Additional file 1: Figure S1. Levels of selected platelet-activating factor and platelet-activating factor-like molecules in the blood of Lassa fever and Ebola patients. [file 12985_2020_1459_MOESM1_ESM.docx]

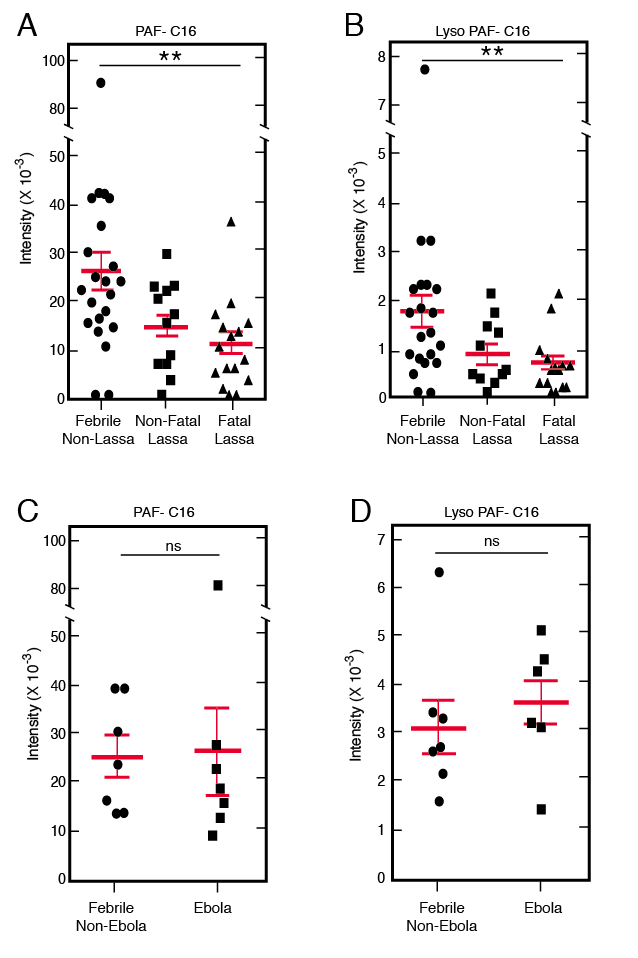


Figure S1. Levels of selected platelet-activating factor and platelet-activating factor-like molecules in the blood of Lassa fever and Ebola patients. Levels of PAF-C16 and lyso PAF-C16 in samples from febrile patients that tested negative for Lassa fever (n=22) were compared to samples from patients with non-fatal (n=12) or fatal Lassa fever (n=16). PAF-C16 and lyso PAF-C16 are not dysregulated in the blood of Ebola positive patients (n=7) compared to febrile controls (n=7). Significance levels (p values from one way ANOVA) are **p < 0.001; ns = non-significant.
